# A Case Report of a Positive Antinuclear Ribonucleoprotein Antibody, a Weak-Positive Antinuclear Antibody, Elevated C3 Complement, and Possibly Trauma-Induced Rasmussen’s Encephalitis

**DOI:** 10.7759/cureus.60415

**Published:** 2024-05-16

**Authors:** Rabiu Momoh

**Affiliations:** 1 Critical Care, William Harvey Hospital, Ashford, GBR

**Keywords:** rasmussen’s encephalitis, autoimmune, radiology and epilepsy, immunosuppressive therapy, head trauma, drug-refractory seizures, seizures, neurology, epilepsy, hemispherectomy

## Abstract

A case of a late-onset Rasmussen’s encephalitis (RE) presenting with drug-refractory focal epilepsy and progressive hemispheric cerebral atrophy noted on a serial radiologic head scan done on a gentleman in his 30s is presented. A positive antinuclear ribonucleoprotein antibody test, a weak-positive antinuclear antibody test, an elevated C3 complement, and possible trauma were identified as potential causative or promoting factors for RE in this patient. Literature evidence regarding the challenges with the aetiopathogenesis description, diagnosis, and management of this rare condition has been reviewed in this article. Exploring an aetiological-based diagnosis of this condition could open research and interventional opportunities into aetiology-guided management opportunities in this condition.

## Introduction

Rasmussen’s encephalitis (RE) was first described by the late Canadian neurosurgeon Theodore Rasmussen in 1958. It is a rare progressive neurological condition commonly affecting children (with a median age of six years). It is associated with chronic inflammation of one hemisphere of the brain causing drug-resistant focal epilepsy, progressive hemiplegia, and problems with cognition. A United Kingdom surveillance study quoted an RE prevalence of 1.8 per million people and an incidence rate of 1.7 per 10 million people under the age of 16 years. A German study revealed an incidence of 2.4 per 10 million persons under the age of 18 years for this condition. A prodromal phase, an acute phase, and a stable disease phase have been described in the progression of RE [1].

The definitive aetiology of RE is largely unknown. Genetic factors, autoimmune disorders, and infective (viral) causes have been suggested in the aetiological description of this condition in the literature [2]. The Sixth European Congress on Epileptology developed a consensus statement on RE, along with the diagnostic criteria and treatment protocol for RE [3]. In this article, a challenging case of a late-onset progressive hemispheric atrophy with drug-resistant epilepsy requiring several hospital admissions (including intensive care unit admissions), and an eventual diagnosis of RE, is described.

## Case presentation

The case of a right-handed 34-year-old male with recurrent hospital and intensive care unit admissions for recurrent drug-resistant seizures with an onset dating back to his early teenage years (age 13) is presented. The only event of note before the seizure onset was an accidental head trauma (hit the right side of the front of his head against a concrete doorframe) with a brief loss of consciousness and no other immediate sequelae. One to two months later, he started to suffer from seizures with loss of consciousness a couple of months before his first seizure onset. Otherwise, he had been a healthy child with normal neurodevelopment and no history of central nervous system infection or febrile convulsions in his childhood. His seizures were of multiple forms and are described in Table 1 below.

**Table 1:** Seizure types in the patient. A textual description of the electroencephalogram (EEG) telemetry study findings is presented in the table.

Seizure types in the patient	Description
Type 1	Type I: Flashing lights in the middle of his visual fields which spread and go to the left and eventually go to the right side. He can then become aware of a smell, his mouth fills with saliva, and he can drool. Then, the left side of his face starts to twitch. Occasionally, he has an aura followed by a severe headache rather than focal motor manifestations. On other occasions, these episodes can generalize into a generalized tonic-clonic convulsion. These episodes were registered on telemetry, and during sleep, one of the attacks was associated with an ictal rhythm at F4, confirming its epileptic nature
Type II	Vacant staring
Type III	Stiffening of the left lower limb, which can be triggered by walking or movement, particularly in direct contact with the floor
Type IV	Irregular shaking of the head and both arms, with the head deviated to the right or left side, and irregular movements of the lower limbs

The patient experienced one type of the above seizures a day but suffered two generalized tonic-clonic seizures a month, most of them provoked by stress. The patient had no family history of epilepsy. He had no substance abuse history.

On examination, he was alert and oriented to time, place, and person. Vital signs (temperature, pulse rate, blood pressure, respiratory rate, and pulse oximetry) were normal. Power was 5/5 across proximal and distal muscle groups in both the right upper and lower limbs. Power in the left lower limb was 5/5, and in the left upper limb was 4/5 in proximal and distal muscle groups. Sensation was intact across all dermatomes bilaterally. The tone was normal globally.

**Figure 1:**
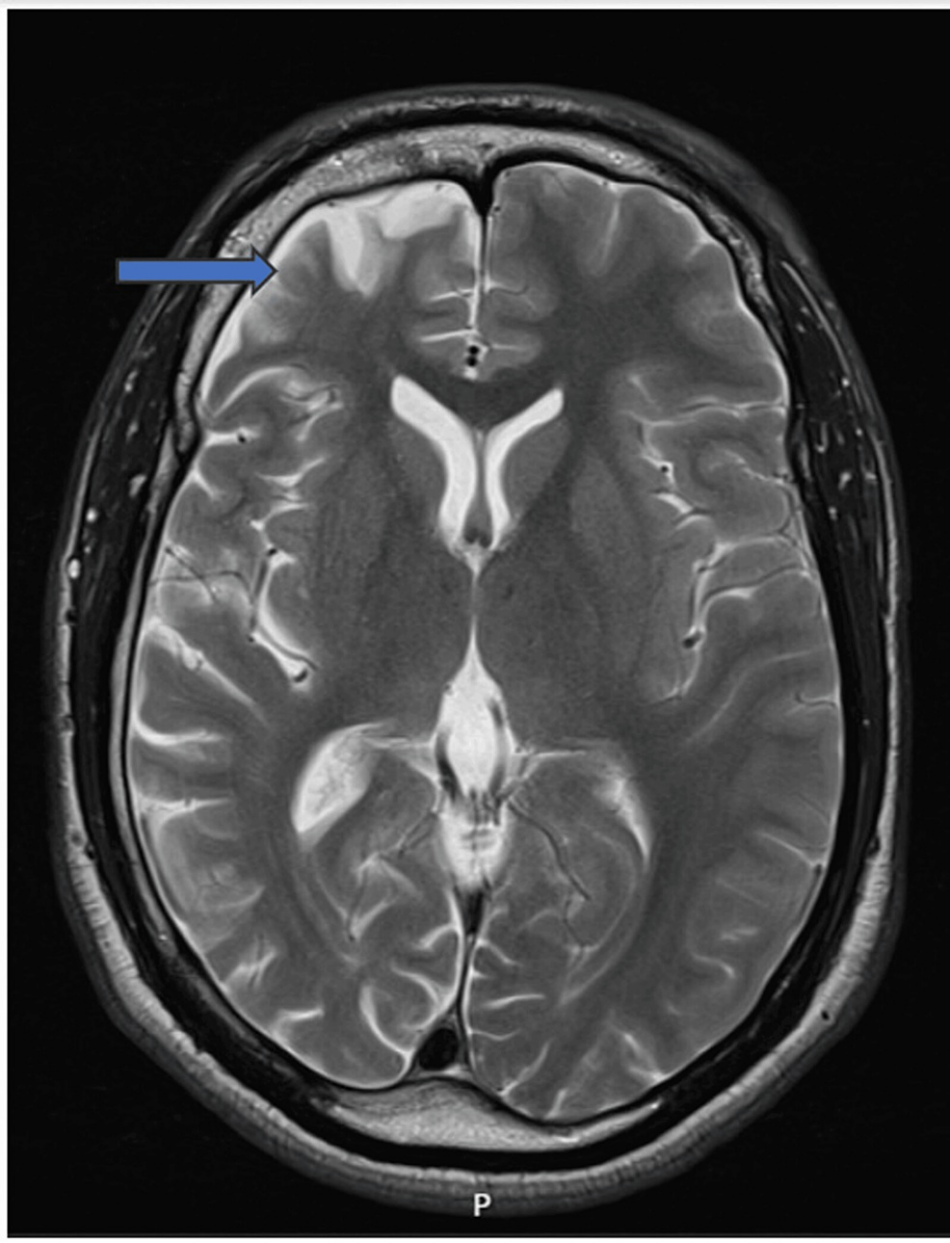
MRI of the head done at age 13 revealing a suggestion of some volume loss in the right frontal lobe with thin gyri and prominent wide sulci and possible gliosis.

MRI of the brain (Figure 1) done in the early period of the onset of his seizures at age 13 revealed some volume loss in the right frontal lobe with thin gyri and prominent wide sulci. A suggestion of possible gliosis of unknown insult or uncertain cortical atrophy affecting the right frontal lobe was made. Elsewhere the brain was unremarkable. No mass effect or midline shift was seen. Particularly, the medial temporal lobes were well preserved on the scan report.

The patient had multiple hospital encounters both as an in-patient (including occasional intensive care unit admissions) and on an outpatient basis for the management of his drug-resistant seizures in the intervening years, and follow-up CT scans in recent years demonstrated progressive atrophy of the right cerebral hemisphere.

**Figure 2:**
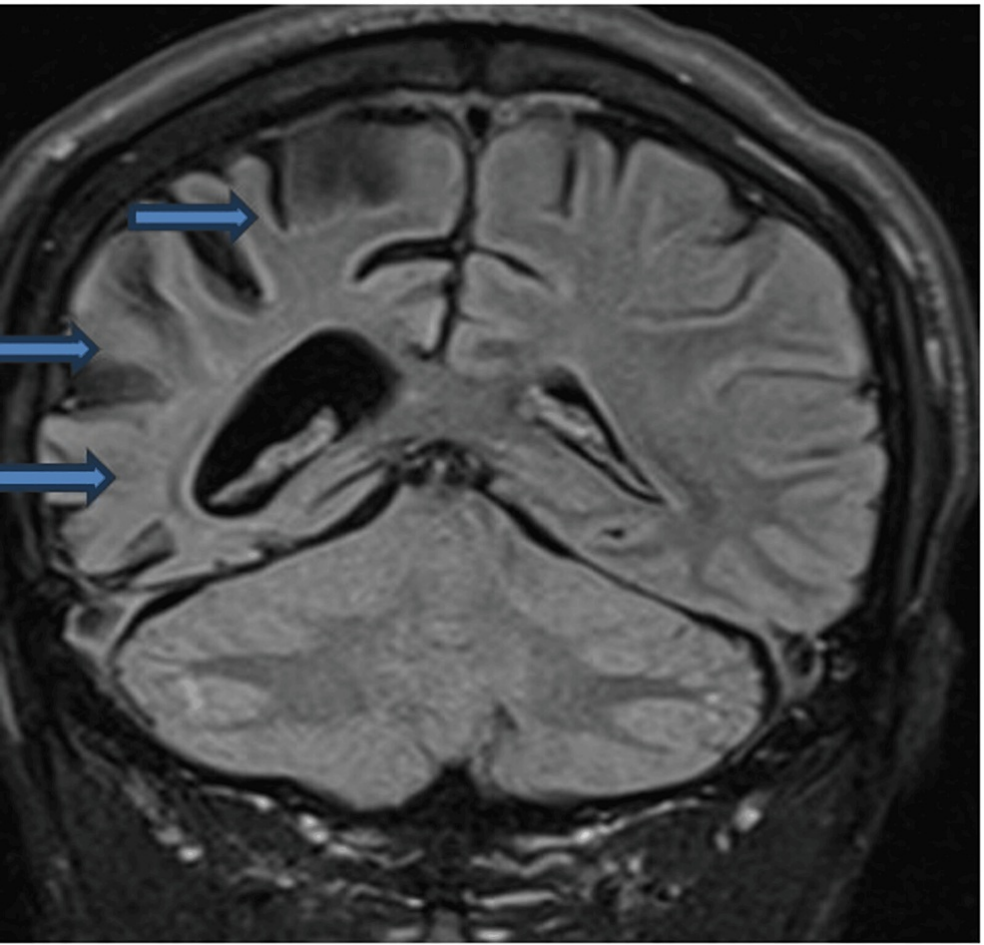
Coronal section of a non-contrast MRI of the head done on the patient at age 33 revealing atrophy of the right cerebral hemisphere.

**Figure 3:**
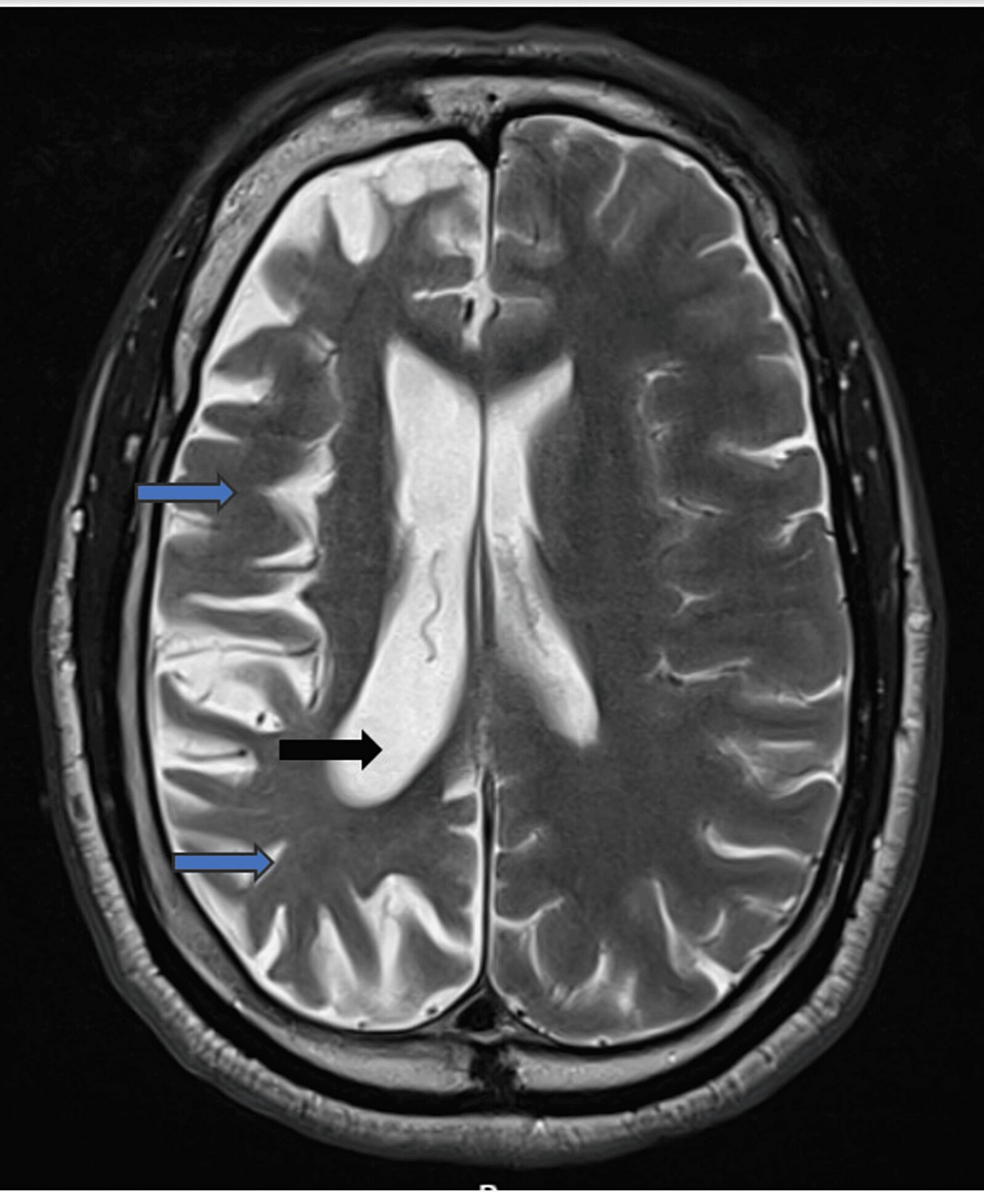
T2/fluid-attenuated inversion recovery MRI of the head axial section revealing right cerebral cortical atrophy (blue arrows) and a comparatively enlarged right lateral ventricle (black arrow).

Non-contrast MRI of the head done one year before this case submission date (Figures 2, 3) revealed no true restriction of the diffusion in the brain parenchyma to indicate acute cerebral infarction or cytotoxic oedema. Longstanding atrophy of the right cerebral hemisphere was noted. No midline shift was seen. Normal vascular flow void of major intracranial arteries consistent with vascular patency was noted. The craniocervical junction and pituitary fossa were unremarkable.

**Figure 4:**
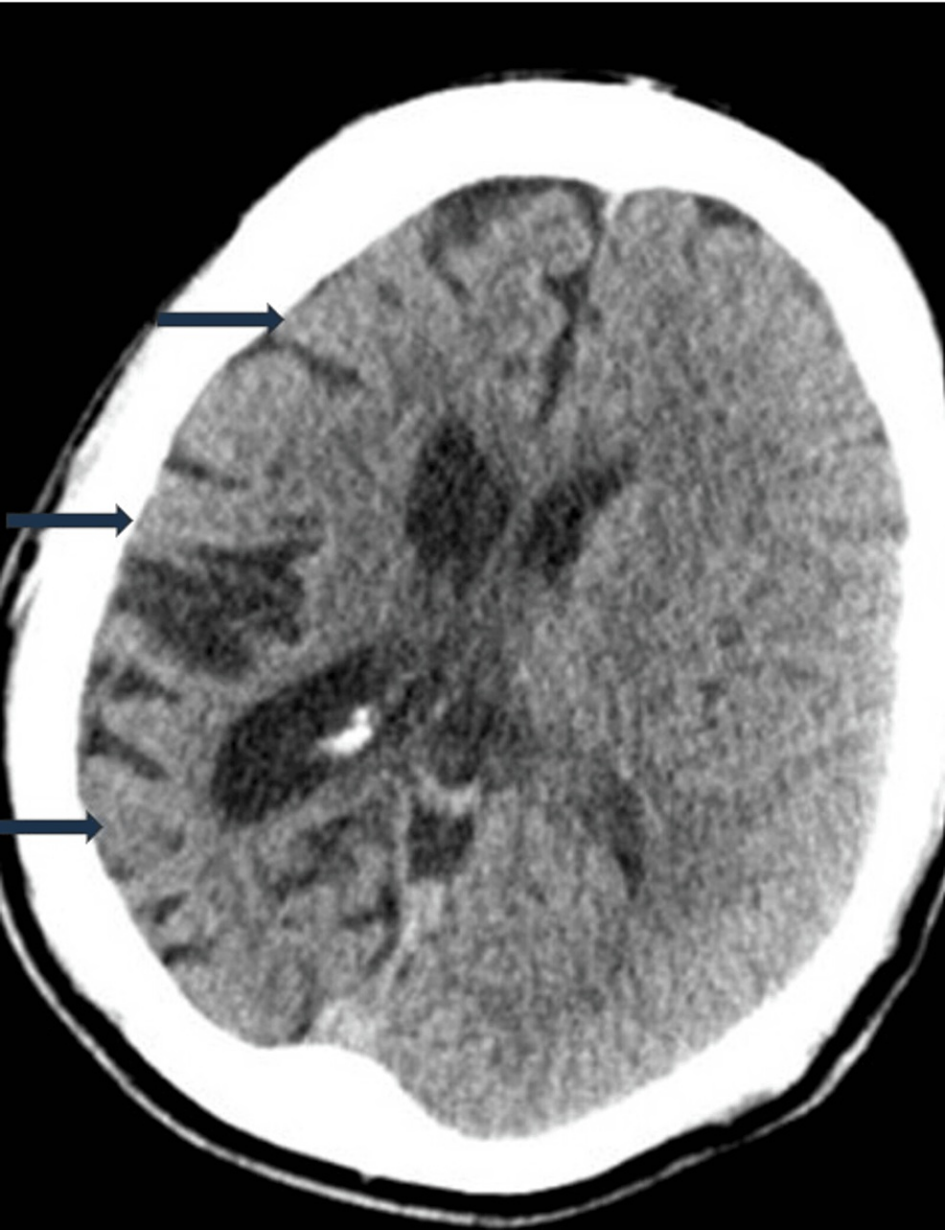
Transverse section of a non-contrast CT of the head revealing a disproportionate atrophy of the right cerebral hemisphere and associated prominence of the sulci and ipsilateral lateral ventricle.

Non-contrast CT of the head (Figure 4) done four months before this case submission on one of the patient’s hospital admissions with generalized tonic-clonic seizures revealed a disproportionate atrophy of the right cerebral hemisphere and associated prominence of the sulci and ipsilateral lateral ventricle. No acute intracranial haemorrhage or large territory infarction was seen. Major basal cisterns were clear. No calvarial or skull base fracture was seen.

The relevant results of the investigations done to assess the patient are outlined in Table 2.

**Table 2:** Relevant results of investigations.

Test	Result/Result comment	Reference
Muscle biopsy study	Normal study	-
Genetic screening: Common *MERFF* (myoclonic epilepsy with ragged red fibres) mutations (m.8344 and m.8363)	Not detected	-
Genetic screening: m.3243A>G mutation	Negative	-
Genetic screening: *POLG1* mutations (p.A467T, m.w.748S and p.G848S, c.1399G>A, c.1760C>T, C.2243G>C, c.2542G>A, and c.2740A>C)	Negative	-
Cerebrospinal fluid (CSF) studies	Normal study with normal CSF lactate	-
Rheumatoid factor screen	Negative	-
Anti-Ro antibody screen	Negative	-
Anti-La antibody screen	Negative	-
Antinuclear antibody screen	Weak positive (1:160–320)	Strong positive (1:>/=640)
Antineutrophil cytoplasmic antibody screen	Negative	-
Anti-double stranded DNA (anti-dsDNA) screen (Phadia method)	1 IU/mL (negative)	0-9 IU/mL (negative); 10–15 IU/mL (equivocal); >15 IU/mL (positive)
Antinuclear ribonucleoprotein (anti-RNP) antibody	Positive	-
Thyroid function test	Normal study	-
HbA1C test	40 mmol/mol	<42 mmol/mol
Random blood lactate check	Normal study	-
C4	0.45 g/L	0.14–0.54 g/L
C3	2.05 g/L	0.75–1.65 g/L
HIV 1 and 2	None reactive	-

The patient had several combinations of anticonvulsants throughout his illness with this condition with recurrent modifications made during clinic attendances and hospital or intensive care unit admissions. He was noted intolerant to the following anticonvulsants: topiramate, levetiracetam, and lacosamide. The relevant latest timeline of the events in the patient’s care is provided below.

Fourteen months before case submission, a diagnosis of RE was formally decided based on the patient’s clinical and radiologic evidence suggesting RE. Based on the Sixth European Congress on Epileptology’s criteria for diagnosing RE, the patient met most of the three-point Part A diagnostic criteria which included the clinical presence of focal seizures and unilateral cortical deficits, the presence of occasional unilateral seizure onset, and the presence of MRI features of unihemispheric focal cortical atrophy. Treatment with pulse intravenous steroid was done and low-dose oral prednisolone 5 mg OD was continued. Oral azathioprine 50 mg BD was commenced in addition to his combination of anticonvulsants at that time. His two-month hospital stay around this period was complicated by a provoked deep vein thrombosis (DVT) for which the patient was initiated on warfarin treatment.

Nine and 10 months before case submission, the patient received two courses of intravenous immunoglobulins (IVIG), 2 g/kg total dose split over five days in each course. He was planned for a third course eight months before this case submission but developed a bilateral pulmonary embolism while still on oral warfarin treatment for his DVT and the third course was held off. His pulmonary embolism was treated with oral edoxaban, as the patient was noted to be poorly compliant with oral warfarin treatment monitoring. A contrast CT of the chest, abdomen, and pelvis done during this period did not reveal any feature to suggest intracavitary malignancy in the body areas studied. He continued follow-up care in the outpatient thrombosis clinic

Five months before case submission: At the patient’s most recent hospital presentation for a status epilepticus, an infective cause of seizure exacerbation was excluded. IVIG five-day course (total dose 2 g/kg) was repeated on this admission with no complications. The patient’s medication list at the discharge point of this hospital admission included PO azathioprine 50 mg BD, PO brivaracetam 100 mg BD, PO cenobamate 250 mg BD, PO clobazam 5 mg BD, PO edoxaban 60 mg OD, PO lansoprazole 30 mg OD, PO phenytoin sodium 200 mg ON, PO pregabalin 200 mg B.D, PO cholecalciferol (400 unit)/calcium carbonate (1.5 g) one tablet BD, and PO high-dose prednisolone 55 mg OD (planned for a reducing regime - drop daily dose by 5 mg every month, and when down to 10 mg, daily dose, followed by reducing the daily dose by 1 mg every month until stopped).

The patient continues to receive joint care from his general practitioner, a district general hospital’s neurology unit, and a tertiary hospital’s neurology unit. He receives community input from his assigned epilepsy specialist nurse. The patient and his next-of-kin were aware of the potential of vagus nerve stimulator therapy and hemispherectomy or its modifications as treatment options for the patient’s condition.

## Discussion

We have described a diagnosis of RE in a 34-year-old male with possibly trauma and/or underlying autoimmune disorder as either an initiating or promoting factor for this rare condition in the patient.

RE was first described in 1958 in a publication by Theodore Rasmussen, Jerzy Olszewski, and Donald Lloyd-Smith. They revealed that chronic inflammation from uncommon histological specimens from three children (with a scarred, atrophic brain) was the cause of their refractory seizures against a previously held neuropathological notion that the patient’s refractory seizures caused perivascular cuffing [4]. RE is largely known to affect one cerebral hemisphere, with drug-resistant seizures, hemiparesis, hemianopsia, and cognitive decline being the major features. The condition is known to affect children more, with a mean age of six years, although this condition has been described in adolescents and adults as well [1]. Vadlamudi et al. (2000) published the case of a 54-year-old female with RE [5]. Castellano et al. (2017) published a case series of three female patients (aged 53, 44, and 47) with adult-onset RE [6].

The exact aetiological description of RE is not fully known. Genetic factors, autoimmune disorders, and infective (viral) causes have been suggested in the literature. Ai et al. (2021) performed a whole-exon genome sequencing of 15 patients with RE and uncovered 31 non-silent single-nucleotide variants affecting 16 genes. The functions of the genes with these single-nucleotide variations were related to nerve cell regeneration, antigen presentation, epilepsy, antiviral infection, and schizophrenia. They asserted they had been the first to provide the first genetic factor demonstration of the cause of RE. They also stated that RE patients may have congenital problems with their adaptive immune responses and are at risk of polygenic abnormal conditions or diseases [7]. Other forms of genetic epilepsy include Angelman’s syndrome, *POLG1* mutation, *CDKL5* disorder, *SLC2A1* (Glut1 deficiency syndrome), ring chromosome 20, and Dup 15q syndrome, among others [8].

In this index case, the patient had a positive antinuclear ribonucleoprotein (anti-RNP) antibody screen, with a medium-positive antinuclear antibody (ANA) screen and an elevated C3. Whitney et al. in their 1999 publication suggested that a complement-dependent process may be involved in RE by demonstrating immunoreactivity for IgG, C4, C8, and membrane-attack complex on discrete patches of cerebrocortical neurons in brain samples of three out of five patients with RE which was not present on the brain samples of five control patients with complex partial epilepsy. They suggested that focal inflammation, neuronal loss, and drug-resistant seizures in some RE patients may be the result of intraparenchymal complement activation triggered by pathogenic antibodies [9].

Kinay et al (2008) published a case of a 17-year-old male patient with RE whose father (a first-degree relative) had a diagnosis of Behcet’s syndrome (an autoimmune disorder) [10]. Amrom et al (2014) published a case series describing four cases of patients with RE who went on to be diagnosed with comorbid autoimmune disorders (with systemic lupus erythematosus (SLE) (with elevated serum anti-double stranded deoxyribonuclease titre (anti-dsDNA)), Hashimoto thyroiditis (with positive serum antibodies to thyroglobulin and thyroperoxidase), Crohn’s disease, or ulcerative colitis found in each of the cases they reported). The case with SLE diagnosis also had elevated ANA and elevated perinuclear antineutrophil cytoplasmic antibodies of 3+ without associated antimyeloperoxidase or antiproteinase-3 antibodies. They suggested that patients with RE may have a shared genetic predisposition to autoimmune diseases [11].

Other autoantibodies that have been implicated in autoimmune epilepsy (types I to XII, respectively) include GluR3B peptide antibodies, NMDA-NR1 antibodies, NMDA-NR2 antibodies, GABA-R antibodies, GAD-65 antibodies, VGKC antibodies, LGI1 antibodies, CASPR2 antibodies, glycine receptor (GLYR), cardiolipin antibodies, beta-2 glycoprotein-1 antibodies, and anti-dsDNA antibodies [12].

The description of trauma as a possible aetiology of RE is very scarce in the literature. Soh et al. (2017) attributed trauma as the possible cause of RE in a case report of a 36-year-old female who had suffered a concussive left frontal head injury two weeks before the development of right upper limb epilepsy partialis continua. Extensive investigations to rule out other possible causes were done before trauma was decided as the most likely inducing factor [13]. In this index case, the patient reported a head trauma at age 13 that was accompanied by a momentary loss of consciousness about two months before the onset of seizures.

Fauser et al. (2022), in their retrospective comparative study that included 160 patients with an RE diagnosis, revealed a female preponderance above age seven at manifestation and that fever-associated onset and perinatal complications were higher in patients with RE than in controls (patients with genetic generalized epilepsy and patients with focal cortical dysplasia Type II) [14].

In a 2005 publication, a consensus statement on the diagnosis and treatment of RE was put forward by the Sixth European Congress on Epileptology. They proposed diagnosing RE with the presence of three features on a Part A table category (comprising clinical presence of seizures (focal) and cortical deficits (unilateral), electroencephalogram (EEG) features of mono-hemispheric slowing (with or without epileptiform activities) and unilateral seizure onset, and MRI features of unihemispheric focal cortical atrophy (with grey or white mater T2/fluid-attenuated inversion recovery hyperintense signal or atrophy/hyperintense signal of the ipsilateral caudate head). If Part A criteria are not fulfilled, they suggested diagnosing with two out of three criteria on Part B which included the clinical presence of epilepsia partialis continua or progressive unilateral cortical deficit(s), MRI features of Progressive unihemispheric focal cortical atrophy, and histopathologic brain biopsy demonstration of T-cell-dominated encephalitis with activated microglial cells and reactive astrogliosis. They suggested limiting the need for a brain biopsy to diagnose RE [3].

Concerning the above diagnostic criteria, the index case under review met most of the three-point Part A diagnostic criteria, i.e., the clinical presence of seizures (focal) and cortical deficits (unilateral), occasional unilateral seizure onset, and MRI features of unihemispheric focal cortical atrophy. A brain biopsy was not done in the index case under review.

Differential diagnosis of RE include Dyke-Davidoff-Masson syndrome (DDMS), Sturge-Weber syndrome (and other neurocutaneous syndromes), hemi-megalencephaly, and unihemispheric cerebral vasculitis [15]. The differential diagnosis of stroke, cerebral vasculitis, multiple sclerosis, Creutzfeldt-Jakob disease, and subacute sclerosing panencephalitis were considered and excluded in this case based on history, laboratory studies, and the absence of associated characteristics on MRI and EEG studies done on the patient [16]. DDMS, with characteristic findings of unilateral cerebral atrophy with ipsilateral calvarial hypertrophy and hyperpneumatization of sinuses, could be of congenital or acquired causes and manifests in the intrauterine or perinatal period. This was not the situation with our index patient. The unique features of hemi-megalencephaly (large unihemisphere with homolateral ventriculomegaly) resulting from partial or total hamartomatous overgrowth of the cerebral hemisphere were excluded from serial MRI studies done on the patient.

**Figure 5:**
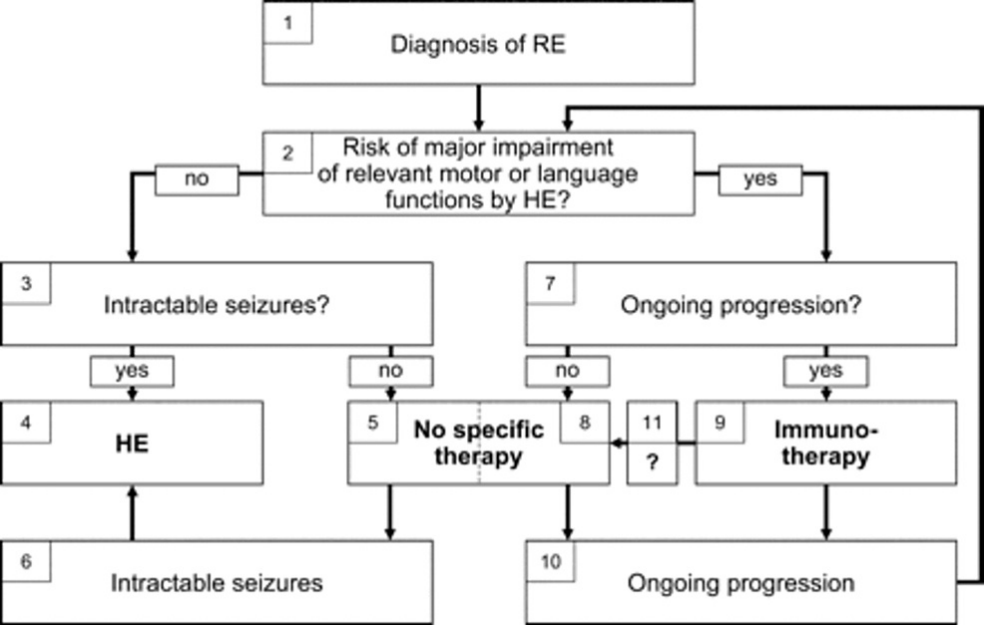
The Sixth European Congress on Epileptology’s proposed algorithm to guide the treatment of Rasmussen’s encephalitis. Permission was obtained to reproduce this algorithm. Reproduced from Bien CG, Granata T, Antozzi C, et al.: Pathogenesis, diagnosis and treatment of Rasmussen encephalitis: a European consensus statement. Brain. 2005, 128:454-71. 10.1093/brain/awh415 [3].

Treatment goals in the management of RE aim to lower inflammation, regain functional ability, and manage/control seizures. Pharmacological treatment (although anticonvulsants are largely not known to be effective in seizure control in RE), immunotherapy (IVIG, steroids, tacrolimus, or a combination), surgical intervention (hemispherectomy surgery and its variants), and rehabilitative procedures are the treatment modalities available for the management of RE. The Sixth European Congress on Epileptology (2005) proposed an algorithm to guide the treatment of RE (Figure 5) [3]. Freeman (2004) suggested that immune-ablative therapy may be the treatment of the future for RE [17].

Jadhav et al. (2023) described the improvement of symptoms with a three-month therapy with IVIG in a nine-year-old male with RE who was later lost to follow-up [18]. Firlik et al. (1999) described the concurrent diagnosis of RE and ganglioglioma affecting the same hemisphere in a four-year-old male with refractory seizures who underwent a functional hemispherectomy with consequent control of seizures [19]. Thomas et al. (2003) described the recurrence of ictal activities on EEG and single-photon emission computed tomography studies done five months post-surgery in a 15-year-old girl who had undergone a functional left hemispherectomy by central disconnection. They suggested that the mechanism that drove the RE in the patient may persist despite this kind of surgery (but may later remit) even if the patient remains clinically seizure-free post-surgery [20].

Casciato et al. (2015), published a case series of five patients (mean age = 22.8 years) with adult-onset RE who underwent surgical intervention. Overall, 80% of the patients studied underwent invasive EEG studies to map the epileptogenic zones. The surgical interventions offered included temporal lobectomy, plus insular and frontobasal corticectomy in one patient, three patients underwent frontal corticectomy, and one patient underwent temporal lobectomy. At their last observation of these cases, they reported two of the cases had rare disabling seizures, one case was seizure-free, one had a moderate reduction in seizures, and one made no clinical improvement [21].

Post-hemispherectomy surgery, most RE patients are left with paralysis, speech impairment, and cognitive dysfunction. Telfeian et al. (2002), however, reported the recovery of language function in a right-handed 16-year-old patient with RE who underwent a left hemispherectomy with the termination of seizures post-surgery [22]. The affectation of the contralateral brain with RE in certain patients post-surgery has been alluded to in the literature [23]. Transcranial magnetic stimulation and vagal nerve stimulator use have been suggested in the treatment of patients with RE [24]. Grujic et al. (2011) revealed a 50% reduction in seizure frequency in a patient with adult-onset RE who had a vagal nerve stimulator implanted [25].

## Conclusions

A rare case of RE in an adult male is presented in this report. A description of combined positive anti-RNP antibody test, weak-positive ANA, elevated C3 complement, and possibly head trauma as inducing factors for this rare condition (RE) has been rarely alluded to in the literature. Multidisciplinary care aspects (from medical teams/neurologists, general practitioners, epilepsy specialist nurses, and intensive care teams) in the management of patients with this rare condition have been highlighted. Neurosurgical intervention such as hemispherectomy and its modifications may be considered in the treatment of this condition. More research efforts into an aetiology-guided treatment approach for this condition are suggested.
